# Liposarcoma With Extensive Necrosis Obscuring the Diagnosis in an HIV-Positive Patient: A Potential Diagnostic Pitfall

**DOI:** 10.7759/cureus.106982

**Published:** 2026-04-13

**Authors:** Ali Khrayzat, Souha Kanj, Samer Deeba, Najla Fakhruddin

**Affiliations:** 1 Department of Pathology and Laboratory Medicine, American University of Beirut Medical Center, Beirut, LBN; 2 Department of Internal Medicine, American University of Beirut Medical Center, Beirut, LBN; 3 Department of Surgery, American University of Beirut Medical Center, Beirut, LBN

**Keywords:** acute inflammation, diagnostic pitfall, hiv infection, liposarcoma, tumor necrosis

## Abstract

Liposarcoma is one of the most common soft tissue sarcomas in adults; however, its occurrence in patients with HIV is rarely reported. High-grade liposarcomas may demonstrate extensive necrosis, which can complicate pathologic evaluation, particularly in limited biopsy material, and may result in misinterpretation as an infectious or reactive process.

We report a 44-year-old HIV-positive man, diagnosed 1 year earlier, with an undetectable viral load, who presented with a deep soft tissue mass initially interpreted on fine-needle aspiration and core needle biopsy (CNB) as an abscess because of extensive acute inflammation and necrosis. Subsequent surgical resection revealed a dedifferentiated liposarcoma (DDLPS) characterized by extensive necrosis and associated acute inflammation, with identification of a well-differentiated component and murine double minute 2 (MDM2) positivity by immunohistochemistry.

This case highlights that extensive necrosis and acute inflammation can represent an important diagnostic pitfall in small biopsies, where malignant components may be obscured or absent. It underscores the importance of clinicoradiologic correlation, adequate tissue sampling, and the use of ancillary testing in the evaluation of soft tissue sarcomas.

## Introduction

Liposarcoma is one of the most common soft-tissue sarcomas in adults, accounting for approximately 13-20% of cases [1]. Although individuals with HIV are at increased risk for certain malignancies, most notably Kaposi sarcoma and lymphomas with established viral etiologies [2], soft-tissue sarcomas are not characteristically associated with HIV infection, and liposarcomas are exceptionally rare in this population [3].

Tumor necrosis is frequently encountered in large or high-grade soft-tissue sarcomas and can be associated with neutrophilic inflammation [4,5]. In limited biopsy samples, such necrotic and suppurative changes may predominate and obscure underlying malignant elements, posing a potential diagnostic pitfall.

Core needle biopsy (CNB) is widely used in the evaluation of soft-tissue masses, but its diagnostic reliability decreases when sampling is limited to necrotic or heavily inflamed regions rather than viable tumor [6]. In these circumstances, biopsy findings may appear benign or nondiagnostic, underscoring the need for careful radiologic correlation, targeted repeat sampling, and adjunctive studies when suspicion for sarcoma remains high. In particular, overexpression of murine double minute 2 (MDM2) serves as a key diagnostic marker for distinguishing dedifferentiated liposarcoma (DDLPS) from benign or reactive mimics.

Here, we present a rare case of DDLPS in an HIV-positive patient, highlighting the diagnostic challenges posed by extensive necrosis and inflammation in core biopsy material. The initial core biopsy suggested a reactive inflammatory process; however, discordance with radiologic findings prompted repeat evaluation and surgical excision, which ultimately led to the definitive diagnosis.

## Case presentation

**Figure 1:**
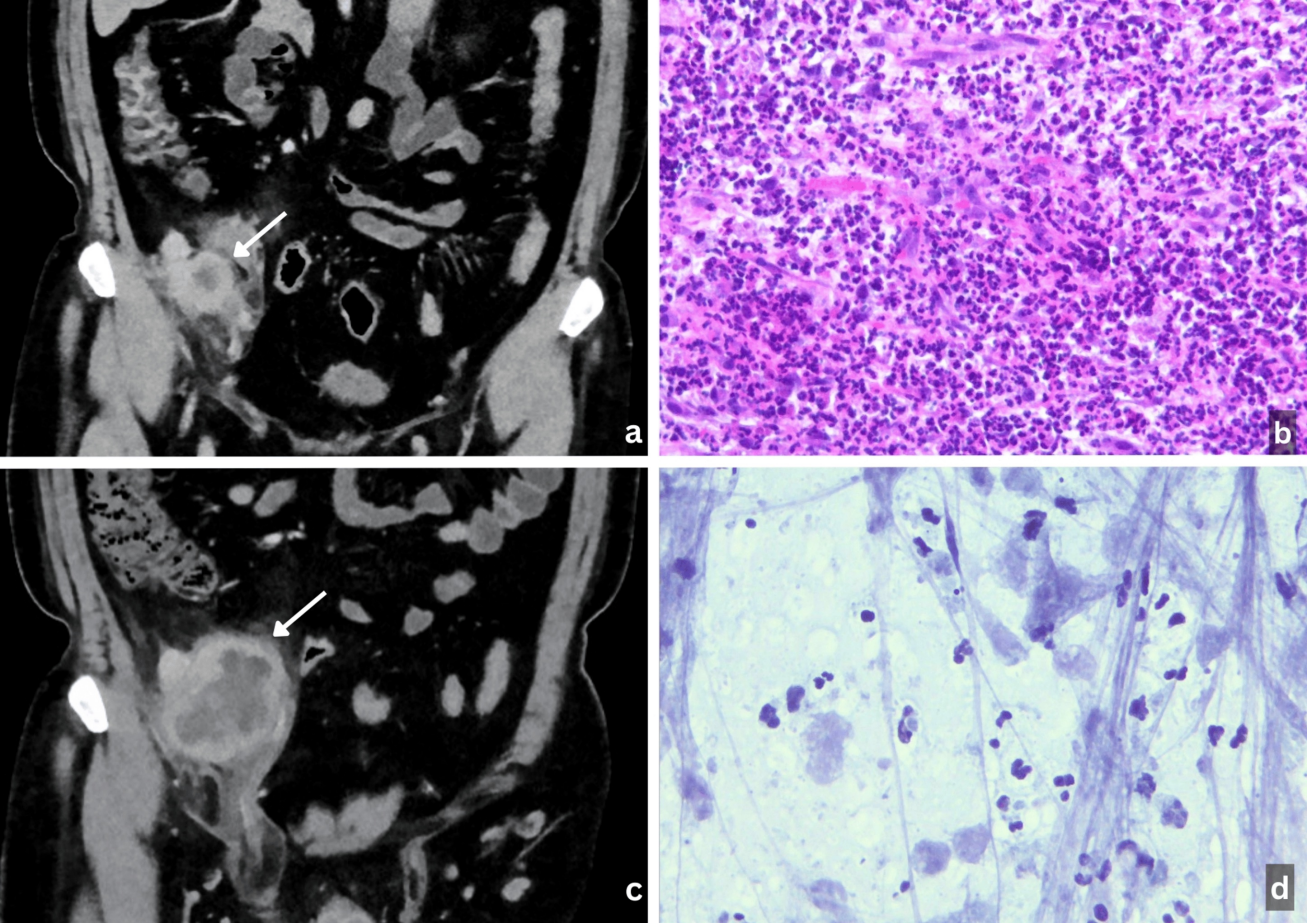
Radiologic and histologic evaluation of the right pelvic/inguinal mass. (a) Initial contrast-enhanced CT scan demonstrating a 3 × 3 cm right pelvic/inguinal soft-tissue mass with early central low attenuation, suggestive of necrosis (white arrow). (b) Core needle biopsy showing predominantly acute suppurative inflammation with abundant neutrophils and granulation tissue; no definitive malignant cells identified (hematoxylin and eosin, 200×). (c) Interval CT scan revealing significant enlargement of the mass, with a more prominent necrotic center and increased surrounding soft-tissue reaction (white arrow). (d) Fine-needle aspiration (FNA) yielding purulent material composed of acute inflammatory cells, without cytologic evidence of neoplasia (Papanicolaou stain, 400×).

A 44-year-old man with HIV infection, diagnosed in 2024, was maintained on bictegravir/emtricitabine/tenofovir. Six months after initiating therapy, he had an undetectable viral load and a CD4 count of 408. In July 2025, he was evaluated for elective right inguinal hernia repair, during which imaging unexpectedly revealed a 3 cm right iliac fossa mass extending through the inguinal canal. CT of the abdomen and pelvis demonstrated a lobulated soft-tissue lesion with surrounding fat stranding and a small necrotic focus tracking into the right hemiscrotum (Figure 1a).

On July 9, 2025, an ultrasound-guided CNB was performed, revealing a prominently vascular and edematous stroma with acute inflammation and granulation tissue, without evidence of malignancy (Figure 1b). Special stains for bacteria, mycobacteria, and fungi were negative, and human herpesvirus 8 (HHV-8) immunostaining did not support a diagnosis of Kaposi sarcoma. A diagnosis of an acute inflammatory process was rendered, based on which empiric doxycycline therapy was initiated for possible lymphogranuloma venereum.

Over the following weeks, the patient developed progressive right groin pain, swelling, and night sweats without fever. A repeat CT scan on July 31, 2025, demonstrated interval enlargement of the right pelvic mass to 7.5 × 6.4 cm, with an expanded necrotic center and further extension through the inguinal canal (Figure 1c). On August 1, 2025, an ultrasound-guided aspiration of the necrotic component yielded approximately 6 mL of purulent material. Cytologic examination revealed acute inflammation with atypical cells interpreted as reactive, with no malignant cells identified (Figure 1d). Despite antimicrobial therapy, the mass continued to enlarge. Of note, bacterial, fungal, and mycobacterial cultures and stains were obtained and later returned negative, as did 16S ribosomal ribonucleic acid (16S rRNA) testing. Venereal Disease Research Laboratory (VDRL) and Chlamydia serologies also returned negative.

On August 15, 2025, the patient underwent excision of the right retroperitoneal mass as well as a separate mass from the right inguinal/spermatic cord region, which was intraoperatively labeled as “lipoma of the cord.” On gross examination, the inguinal mass measured approximately 9.5 × 7 × 2.5 cm and consisted of lobulated tan-yellow adipose tissue with focal firm, fibrous areas. The retroperitoneal mass measured 13 × 10 × 6 cm and was partially encapsulated. The cut surface was variegated, showing extensive central tan-yellow necrosis and focal hemorrhage, surrounded by peripheral zones of firm gray-white tissue. Representative sections from different regions were thoroughly sampled for histologic evaluation.

**Figure 2:**
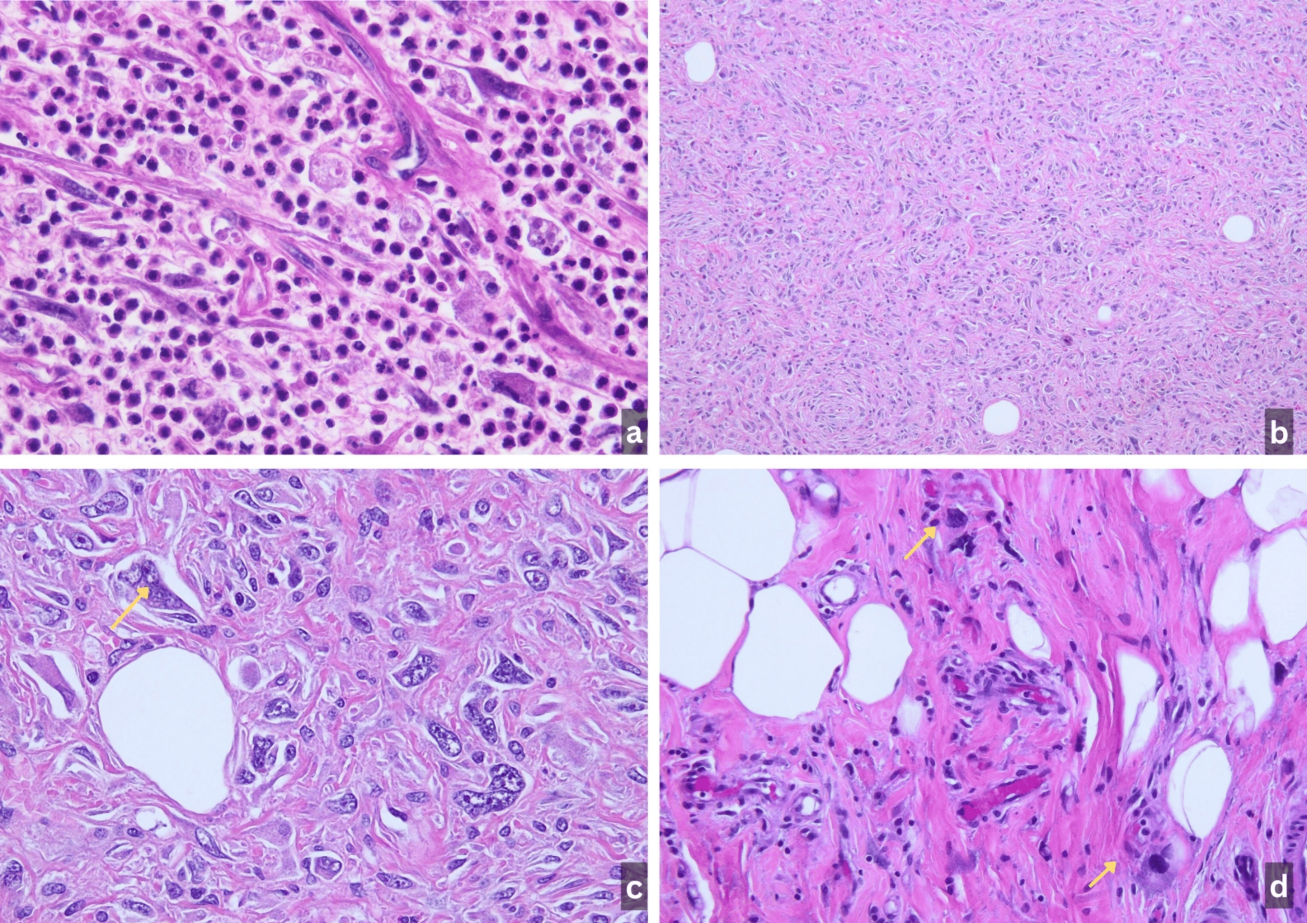
Histopathologic examination of the resected masses. (a) Area of tumor showing morphology similar to that of the core needle biopsy, with predominant acute inflammation (H&E, 400×). (b) Low-power examination of a solid high-grade component demonstrating sheets of atypical spindle to pleomorphic cells (H&E, 100×). (c) Higher-magnification view of the dedifferentiated high-grade sarcoma exhibiting marked nuclear atypia and pleomorphism (yellow arrow) (H&E, 400×). (d) Area from the well-differentiated liposarcoma (WDLPS) component showing mature adipocytic proliferation with atypical hyperchromatic stromal cells (yellow arrows) (H&E, 400×).

Microscopically, the inguinal/spermatic cord specimen demonstrated features consistent with well-differentiated liposarcoma. The resected primary mass revealed extensive areas of acute inflammation and necrosis, closely resembling the findings seen in the prior biopsies. Scattered foci of viable tumor showed a high-grade sarcomatous proliferation. Based on these findings, a diagnosis of DDLPS was made for the primary mass (Figure 2). Immunostaining for MDM2 was positive in both the inguinal and primary mass specimens, confirming the diagnosis. Resection margins were free of tumor, and the postoperative course was uneventful.

## Discussion

This case describes a rare presentation of DDLPS characterized by extensive necrosis and acute inflammation in a patient with HIV. It highlights a critical diagnostic pitfall: biopsy specimens dominated by necrotic debris and neutrophil-rich inflammation can obscure malignant cells, leading to misinterpretation and diagnostic delay. Although such necrosis is a recognized feature of high-grade sarcomas, its occurrence in an HIV-positive individual presents a significant diagnostic challenge by mimicking opportunistic infection. A pivotal turning point in this case was the rapid interval enlargement of the mass despite empiric antimicrobial therapy. This striking radiologic-pathologic discordance necessitated surgical excision, guided by the clinical suspicion that the initial needle biopsies were nondiagnostic because of sampling error.

HIV infection is characterized by a paradoxical state of adaptive immunosuppression and chronic innate immune activation [7, 8]. The depletion and functional exhaustion of CD4+ T cells, CD8+ cytotoxic T cells, and natural killer (NK) cells compromise the host’s capacity for tumor surveillance, allowing malignant cells to evade detection [2]. Conversely, innate components such as neutrophils and monocytes remain chronically active; these cells are recruited to the tumor microenvironment by pro-inflammatory signals, contributing to the exuberant inflammatory response observed histologically [5]. Together, these mechanisms may provide a permissive physiological environment for the de novo development of rare non-AIDS-defining malignancies, such as liposarcoma, even in the absence of a direct viral oncogenic driver [2, 3].

Extensive tumor necrosis is a well-recognized feature of high-grade soft tissue sarcomas and is particularly common in aggressive or rapidly growing lesions [9]. When necrosis predominates, tissue sampling, especially by CNB, may yield only necrotic debris with little or no viable tumor [6]. Tumor necrosis can also elicit a secondary sterile inflammatory response through the release of intracellular damage-associated molecular patterns from dying cells, activating innate immune pathways and recruiting neutrophils even in the absence of infection [10]. Experimental studies have further demonstrated that necrotic tissue can trigger inflammasome-mediated inflammatory signaling, promoting a neutrophil-predominant acute inflammatory response [11].

Liposarcoma occurring in HIV-positive patients is exceedingly rare. To our knowledge, only a limited number of individual cases have been reported in the literature, including mediastinal liposarcoma [12], cervicothoracic liposarcoma [13], and a giant retroperitoneal liposarcoma causing renal displacement [14]. In a systematic review of sarcomas arising in immunodeficient conditions, Bhatia K et al. identified only sporadic reports of liposarcoma among HIV-infected individuals, underscoring the exceptional rarity of this entity in this population [3].

## Conclusions

In summary, we report a case of DDLPS in a patient with HIV infection that initially presented as a necrotic, inflammatory mass and was repeatedly interpreted on limited biopsy material as an infectious process. Extensive tumor necrosis and acute inflammation obscured the malignant component, resulting in delayed diagnosis despite radiologic progression. This case underscores that, in the setting of discordant clinicoradiologic and histologic findings, particularly when biopsy samples demonstrate only necrosis or inflammation, persistent clinical suspicion and repeat or more extensive sampling are essential. Awareness of this diagnostic pitfall is critical to prevent delayed recognition and management of aggressive soft tissue sarcomas.
